# Why do open and distance education students drop out? Views from various stakeholders

**DOI:** 10.1186/s41239-022-00333-x

**Published:** 2022-06-03

**Authors:** Ayşe Bağrıacık Yılmaz, Serçin Karataş

**Affiliations:** 1 Computer Education and Instructional Technology, Egitim Fakultesi, Aydın Adnan Menderes University, No: Z25, 09000 Aydın, Turkey; 2 Computer Education and Instructional Technology, Gazi Egitim Fakultesi, Gazi Universitesi, Bosna Binası No: 329, Besevler, 06500 Ankara, Turkey

**Keywords:** Dropout, Retention, Open and distance education, Online learning

## Abstract

While the demand for open and distance education is increasing, it also faces high dropout rates. The reasons and solutions for student dropouts need considerable attention. This study aims to uncover the reasons for student dropouts considering the perspective of students, field experts, instructors, administrators, and support staff. Data from semi-structured interviews with 40 participants revealed that students decide to drop out mainly due to four main reasons: internal reasons, external reasons, student characteristics, and student skills. These reasons include 37 sub-factors such as academic integration, social integration, financial status, personality, and self-regulation. The sub-factors and selected quotations from the interviews are presented in the findings. According to the results, administrators, field experts, instructors, and support staff are not aware of all the dropout reasons. The results of this research are believed to guide researchers, practitioners, and administrators in enhancing the quality of open and distance education.

## Introduction

Open and distance education (ODE) is growing rapidly and in high demand (Muljana & Luo, 2019). The number of students enrolled in distance education at the undergraduate and graduate levels increases every year. The growth between 2012 and 2016 was 17.2% as reported by Allen and Seaman (2018). While the increase in the number of students continues, ODE experience low retention and completion rates (Muljana & Luo, 2019; Radovan, 2019; Xavier & Meneses, 2020) with higher dropout rates than those of face-to-face learning (Gaytan, 2015; Quayyum et al., 2019; Radovan, 2019).

Dropping out is a multidimensional phenomenon (Grau-Valldosera & Minguillón, 2014; Li & Wong, 2019) that might be explained through various reasons affecting students’ drop out decision. There are various studies in the literature to determine why learners drop out. These studies sometimes address the situation from a holistic perspective and sometimes in a narrower framework. Since dropping out relates to financial damage and loss of prestige for institutions (Reissman, 2012), it should be considered not only by researchers but also by educational institutions.

One leading factor for dropouts highlighted in the literature is the interaction that has become known in the literature through the research of Moore (1989). As a matter of fact, Moore (1989) argued that education without interaction would consist only of transferring knowledge. Student–instructor interaction (Gaytan, 2015; Shikulo & Lekhetho, 2020; Sorensen & Donovan, 2017), social interaction (Boton & Gregory, 2015; Hawkins et al., 2012) and student–student interaction (Muljana & Luo, 2019; Stone & O’Shea, 2019) are associated with drop out decisions. Student–instructor interaction constitutes an important dimension of the interaction. In addition to the interaction with the instructor, the qualifications of the instructor are also effective in the drop out decisions (Thistoll & Yates, 2016). The way the instructors address their students in emails can even affect students’ decisions (Stone & O’Shea, 2019). Hence institutions should be extremely careful in the selection of instructors (Özcan, 2019).

Another significant point for institutions is the extent to which the qualifications meet student expectations. The quality of educational and technical support provided by the institution is important in drop out decisions (Gaytan, 2015; Muljana & Luo, 2019). Moreover, orientation programs influence the rates of dropping out (Arhin & Wang’eri, 2018). When orientation programs are not organized effectively, students experience uncertainties and might prefer to give up instead of facing the difficulties.

Student characteristics and skills that students have before enrolling in the system is another dimension that needs to be considered when dropping out is mentioned (Lakhal & Khechine, 2021; Reissman, 2012; Rovai, 2003). Marital status, status of employment, gender (Cochran et al., 2014; Li & Wong, 2019), age (Muljana & Luo, 2019), self-efficacy, online learning preparation (Yükseltürk et al., 2014), autonomy (Verdinelli & Kutner, 2016) might influence students’ decisions. Besides, time management, self-regulation (Muljana & Luo, 2019; Tubilleja, 2019; Watts, 2019), and metacognitive thinking (Poitrast, 2016) are among the student skills observed in dropout studies.

Numerous factors might affect the decision to drop out and these factors are not limited to those mentioned here. An effective way to understand the reasons for student dropouts is to ask the student herself/himself. Several qualitative studies focused on the opinions of students (Aydin et al., 2019; Budiman, 2018) and/or instructors (Gaytan, 2015) to understand dropout reasons. However, as students are not the only stakeholders of ODE, it will be useful to reach as many stakeholders as possible in order to examine the problem more comprehensively. The researchers in this study analyzed the situation in detail by involving ODE experts, instructors, administrators, and support staff in addition to students.

Another reason that encourages researchers to conduct this study is that although there is a Giga University [with more than 1 million students (Bozkurt, 2019)] in their country, the number of drop out studies sourced from that country has been insufficient. This Giga University (Anadolu University) has more than 3 million ODE students, and more than half of these students are passive (Anadolu University, 2019). The number of students dropping out ODE in Turkey is reported to be increasing (Okur et al., 2019) that highlights the need to understand the dropout reasons. The results of a comprehensive study are believed to contribute to not only the national but also the international literature.

### Research question

This study seeks to answer the following question: “Why do open and distance education students drop out?” The researchers tried to understand the reasons through the eyes of various stakeholders.

### Open and distance education (ODE)

The concepts of distance education and open education have been used either separately or together. These two terms have also been used interchangeably. Distance education is a system where the instructor and the student are mostly in separate places during the learning-teaching processes, therefore interact through, various communication technologies (Moore & Kearsley, 2011). UNESCO (2016) defined distance education as a form of education in which the instructor and the student are separated in time and space, through forms of online learning, blended learning, with the use of printed resources delivered to students by mail or other tools.

Wedemeyer (1981) defines open education as providing part-time learning opportunities for students at a distance, who operate with a degree of autonomy and self-direction, but with open mediated access to learning without conventional prerequisites for acceptance or accreditation. According to Rumble (1989) and Maxwell (1995), openness is an approach, and every form of education has a level of openness. Distance education systems are generally considered to be open systems, because students are not required to study in a specific place and time.

The Open University considers open education an approach as well. The Open University states that students have flexible access to the study materials, and they can engage in the activities and assignments from wherever they want. This flexibility and accessibility is identified as “openness” (The Open University, 2019). The openness of a system for students deserves attention to analyze the different educational models.

To clarify, this study will refer to the model as “open and distance education” to specify the point of investigation. This concept is preferred since two of the participating institutions are open education institutions and two are distance education institutions.

These two types of institutions differ in terms of their admission requirements and the course delivery. Undergraduate students are not required to register to the open educational institutions. In distance education institutions, on the other hand, students are asked to provide the required exam scores before they register. While courses in open education institutions in our country are mostly delivered asynchronously using printed sources; courses in distance education institutions are conducted mainly synchronously.

This study prefers to use *open and distance education* instead of *open and distance learning* as only the students officially registered at an institution were included in the study. Specifically, this study focused on higher education students who were *educated* at some institutions, not *learners* who were educated through massive open online courses (MOOCs) or open educational resources (OERs).

## Background of the study

### Defining the concept: drop out

Dropout is defined contrarily by researchers and there is no consensus about its meaning. According to Martinez (2003) dropping out refers to a student leaving the course without ever returning. Similarly, Botelho et al. (2019) define the dropout student as the one who will never return to complete the course. On the other hand, Kember (1995, p. 21) considered the dropout students in five different categories: “(1) non-starters, (2) informal withdrawals who stopped working on the course, (3) formal withdrawals who completed an official procedure, (4) academic failures, and (5) non-continuers who may never have intended to complete a full program of academic study.”

Researchers (Aydin et al., 2019; Stiller & Bachmaier, 2017), generally do not seek the condition that students have officially dropped out of education. Passive students are also often included in the research. In this study, the requirement that students have dropped out using formal procedures is not considered. Students’ own statements are taken as a basis to verify their condition of being a dropout.

### Models for dropout

ODE dropout models are often based on models of face-to-face education. The Student Integration Model, in which Tinto (1975) focuses on why students drop out of face-to-face education, has guided other models. Tinto focuses on student characteristics, academic integration, social integration, goal commitment, and institutional commitment. Furthermore, the student’s academic performance and mental development can also affect the drop out decision.

According to Bean and Metzner’s (1985) *conceptual model of non-traditional student attrition,* characteristics of students, academic variables, environmental variables, academic success, and psychological outcomes affect the drop out decision. The main difference in the reasons for non-traditional (distance education) students and traditional (face to face) students to drop out is that non-traditional students are more affected by environmental factors than social integration factors (Bean & Metzner, 1985). Students with low academic success are expected to have higher dropout rates than those with high academic success. Nevertheless, even if a student has a high academic grade point average, they may drop out if the perceived benefit is low.

Kember (1995) has developed a model of student progress in open learning courses based on Tinto’s (1975) model by stating that distance education students are adult students with different characteristics than those who receive face-to-face education. According to this model, students with a limited educational background are less likely to develop a study approach in line with the requirements of higher education than other students. Besides, ODE students are generally employed and have family responsibilities. Accordingly, a student who can balance family, work, and social life with their studies is more likely to complete the course. In addition, the ratio of time, effort, and cost might affect the student’s decision to continue.

In Rovai’s (2003) well-known model, variables are evaluated in two stages as *prior to admission* and *after admission*. He mentioned student characteristics and skills in prior to admission stage. In the after-admission period, factors related to the student’s work and family, and economic situation are included. Rovai incorporated variables emphasized by Tinto (1975), and Bean and Metzner (1985), and highlighted student needs, learning styles, and teaching styles that were not mentioned previously.

This study is based on Rovai’s model to perform the deductive analysis of the data. For clarity purposes, explanations of some concepts are given below.

### Internal factors

Internal factors, according to Rovai’s model, include all variables “related to education” after admission (Rovai, 2003). These factors include academic integration, social integration, accessibility, goal commitment, and institutional commitment. Tinto (1975) stated that academic integration can be measured by grade performance or intellectual development. Social integration, on the other hand, covers student interactions with the instructor, peers, and administrative staff. Rovai (2003) added accessibility to the composite persistence model considering the work of Workman and Stenard (1996), and associated accessibility with students’ access to information about the institution, educational programs, and courses, and quick e-mail correspondence. The impact of accessibility on persistence is still the subject of studies (Muljana & Luo, 2019). Student commitment to the goal can be measured in terms of educational plans, educational expectations, or career expectations. Goal commitment and institutional commitment together affect the student’s decision to drop out. Academic integration of the student contributes to the goal commitment, and social integration contributes to the institutional commitment (Tinto, 1975). In this study, it would be appropriate to explain some variables that are not mentioned in the models related to dropout/persistence in the literature but are considered as internal factors. Resources refer to any written, printed, and/or electronic material that students use to complete the course. The quality, perceived usefulness, and availability of resources ensure persistence (Li & Wong, 2019). In addition, student-content interaction in open and distance education is a recognized significant phenomenon (Anderson, 2003; Moore, 1993).

The instructor was included in Tinto’s model (1975) with reference to his/her interaction with the students; however, the characteristics of the instructor were not emphasized. As previously mentioned, the literature suggests that some qualifications of the instructor may affect the students’ dropout decisions. In this study, instructor characteristics express a multidimensional concept that includes features such as belief/prejudice towards open and distance education, the reason for teaching, caring for the lesson, digital literacy, open and distance education knowledge and experience, subject matter knowledge, and teaching method.

In the study, flexibility represents the “structure” expression that Moore (1993) stressed. Accordingly, the structure is concerned with the ability of an educational program to adapt or respond to the individual needs of students. An education program should neither make the student very comfortable by not setting any rules nor should be in a very rigid structure.

Another concept that is not encountered in the models is exams. In this study, the effect of the exams refers to factors such as availability of alternative exams (makeup exam, etc.), reliability, and validity of the exams. Exam conditions are also observed to affect student decisions (Okur et al., 2019). Aydin et al. (2019) stated that students consider open and distance education courses easy to complete, referred to as “Perceived ease of completion” in this study. The fact that the students do not want the wrong answers to eliminate the correct answers, which is one of the measures taken for reliability and validity in exams, can also be described as an easy completion expectation.

### External factors

According to Rovai’s (2003) model, external factors consist of all the non-education-related variables in the process after the admission to the system. Rovai lists finance, hours of employment, family responsibilities, outside encouragement, opportunity to transfer, and life crises as external factors. In this study, the hours of employment forms a part of “Business life”. Factors such as intense or flexible work life, the mental comfort of being employed, and legal procedures related to business life.

The financial situation of the person or the amount of the required tuition fee shapes the financial reasons. Family life consists of factors such as the responsibility of children, pregnancy, and marriage. Outside encouragement is considered as either external support or obstruction in this study; because people in a student’s life (spouse, friend, parents, etc.) may not always be encouraging.

Life crises were evaluated in the same sense as Rovai’s (2003) study. Accordingly, situations such as divorce, loss of job, and sickness may cause the student to drop out of education (Tinto, 1975). Opportunity to transfer refers to the possibility of the student to transfer to a different university. If there is no better option, the probability of dropping out may decrease. Social life relates to the student's inability to devote time to his/her education due to the intense social life.

### Student characteristics

Student characteristics are considered in Rovai’s (2003) model as age, ethnicity and gender, intellectual development, academic performance, and academic preparation. In this study, personality structure, belief/preconception, self-suitability, un/consciousness, and technical equipment facilities variables were also added. In addition, study habits and goal commitment, which are under internal factors in Rovai’s model, were evaluated as student characteristics in this study.

Goal commitment includes the psychological orientations of the individual brought to the system. These orientations will determine the way the student interacts within the system (Tinto, 1975). If a student is committed to his goal, s/he will be more likely to continue education (Lee et al., 2013). Personality structure includes the characteristics of the student such as self-confidence, perfectionism, patience, perseverance, and responsibility.

Academic performance, which Rovai (2003) considered as a student characteristic, was associated with academic integration under internal reasons in this study. As a matter of fact, Tinto (1975) stated that academic integration can be measured with grade point average (GPA), as an indicator of academic performance. Academic preparation is considered as the academic background in this study, and it refers to the status of previous university experience, the status of study experience in open and distance education institutions, and the level of knowledge about the program enrolled. It is known that students’ previous experiences, such as having had the drop-out experience, affect their decision of whether to continue their studies or drop out (Aydin et al., 2019; Cochran et al., 2014).

Belief/preconception represents the student’s (usually negative) thoughts about open and distance education. Un/consciousness is about whether the student is conscious about the system and his/her program. For example, students sometimes think that an unfavorable decision taken on the national level was made only by the institution where they studied, and they may distance themselves from the institution. Finally, since open and distance education can no longer be thought of without technical equipment, students’ possession of this technical device may affect their decision to drop out.

### Student skills

Rovai (2003) included computer literacy, information literacy, time management, reading and writing, and computer-based interaction in student skills. In this study, computer literacy and computer-based interaction were considered as digital literacy components and they were associated with the student’s ability to use hardware and software tools at a level that would enable them to learn. The fact that students’ lack of digital competence for learning purposes may cause them to drop out (Bawa, 2016).

Time management is combined with the broader concept of self-regulation. Self-regulation in this study covers the ability of students to carry out their learning processes independently and to manage their time. Self-regulation is a variable emphasized in numerous studies (Aydin et al., 2019; Bawa, 2016; Stiller & Bachmaier, 2017) that affects students’ decision to drop out. Finally, reading and writing is a requirement for literacy and content comprehension prior to the tertiary education, reading and writing skills included in Rovai’s (2003) model were not evaluated in this study.

## Methodology

An answer to the research question was sought using the qualitative research model. Because a complex picture of a certain situation/problem has been tried to be created, which is one of the main features of qualitative research. Qualitative research involves including different perspectives and identifying many factors involved in a situation (Creswell, 2009). Moreover, the event is seen from the perspective of the participant, not the researcher, by focusing on how people interpret their experiences and give meaning to these experiences (Merriam, 2015). Similarly, the phenomenon of dropping out is viewed from the eyes of the stakeholders.

This research is based on the case study design; because the purpose of the case study is to obtain comprehensive, systematic and in-depth information about the situation under consideration (Patton, 2014). Deep and comprehensive information is gathered to understand why open and distance education students drop out of education. Yin (2018) categorized case studies into three as exploratory, descriptive and explanatory case studies. In descriptive case studies, data are presented in a cause–effect relationship. Similarly, this study is based on Yin’s (2018) descriptive case study type to investigate the reasons for students to drop out of open and distance education.

### Study group

Participants consist of 40 people, including 17 dropout students, 5 experts in ODE, 6 instructors, 4 administrators, and 8 support staff. The study was conducted in four well-known ODE institutions in Turkey. Both criterion sampling (Merriam, 2015) and convenience sampling (Patton, 2014) methods were used. The criterion was that students had left an associate or undergraduate program within the last 5 years. In addition, easily accessible participants were interviewed.

**Fig. 1:**
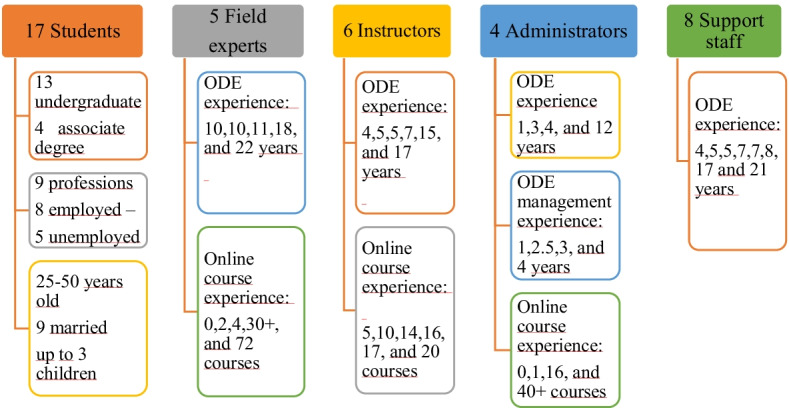
Participant information

The dropout student group includes eight open education students and nine distance education students. In Turkey, one of the most important differences between these two models is that synchronous online classes are not yet common in open education. Moreover, the admission requirements for open education institutions are more flexible than distance education institutions. This research uses the internationally accepted concept of ODE by including students from both groups. Detailed information about the participants is provided to increase the reliability of the research (Fig. 1).

In addition to the students, the opinions of the instructors (6 participants) were consulted. Since many factors influence students’ drop out decisions, from the interaction of the instructors with the students, their qualifications (Thistoll & Yates, 2016), and even the way they address the students (Stone & O’Shea, 2019). Besides, being a stakeholder with high levels of interaction with students, the instructors are believed to provide extensive information and insight on the reasons for student drop-out.

Another group that was thought to provide rich data was field experts. While including the field experts, the criteria was having conducted academic studies on the subject of dropout in open and distance education. In addition, all except for one of the field experts have experience in teaching via distance education.

Finally, administrators and support staff were included in the study. Support staff forms the first contact person for students to ask for help or advice when they have a problem. In fact, in this study, the researchers observed that the students were more comfortable telling the support team about some of the problems that they could not express to the instructors. The administrators, were examined in the study, considering that they would contribute with their observations and experience about the drop-out, as they are responsible for solving the institutional and pedagogical problems and for offering quality education.

Participants were coded as follows:

Institution (I) + Institution order (1, 2, 3,4) + Participant type (S-Student, FE-Field Expert, A-Administrator, Ins-Instructor, SS-Support Staff + Participant order (1, 2, 3, 4…).

Ex.: I2S3; The third student who has left the second institution.

### Data collection

Different semi-structured interview forms for each participant group were prepared based on the literature and field experts’ (8 distance education field experts and 2 qualitative research experts) opinions. Interview is chosen as a data collection method as it allows to see the events through the eyes of the participants (Merriam, 2015; Patton, 2014). Before the original interview form was created, the draft interview form was piloted by implementing it to the participants from each group. In the interview form, there are 21 questions about the reasons of dropping out. These questions were organized around factors such as instructor, interaction, learning resources, accessibility of the institution, orientation programs, and exams in accordance with the literature. A few examples of these questions are given below:

- What are the instructor related factors that cause you to drop out of education?
- How was your interaction (if any) with other students in the course or extracurricular environments (Facebook, WhatsApp etc.)? What are the effects (if any) of this interaction on your decision to drop out?

As previously stated, students who dropped out of open and distance education, instructors, distance education field experts, open and distance education administrators and student support staff were included in this study. Therefore, the above questions were adapted to other participating groups. An example of the instructor questions is as follows: ‘Does your institution inform you about the retention and dropout status of students?’ One of the questions administrators were asked is: ‘What kind of measures do you take as an institution to increase retention rates and decrease dropout rates?’.

Prior to interviews, ethical review and approval was granted from the university where the researchers work. Using this official approval, data were collected from four different universities. Researchers contacted the participants through social media groups; often interested participants directed others to the researchers.

All interviews were conducted by one of the researchers. It took a year to complete the interviews mainly due to difficulty in accessing the dropout students. All participants were volunteers to be interviewed. Most of the participants were interviewed on the phone as per participant’s request. On the other hand, some participants preferred video conferencing and written communication. The researcher recorded the interviews having granted the permission of the participants.

### Data analysis

There are two different methods in qualitative data analysis: descriptive analysis and content analysis. In descriptive analysis, predetermined codes are not exceeded (Yıldırım & Şimşek, 2013). However, since this research cannot be limited to certain codes, content analysis was used in the study mainly by coding, identifying themes, organizing data according to codes and themes (Miles & Huberman, 1994).

**Fig. 2:**
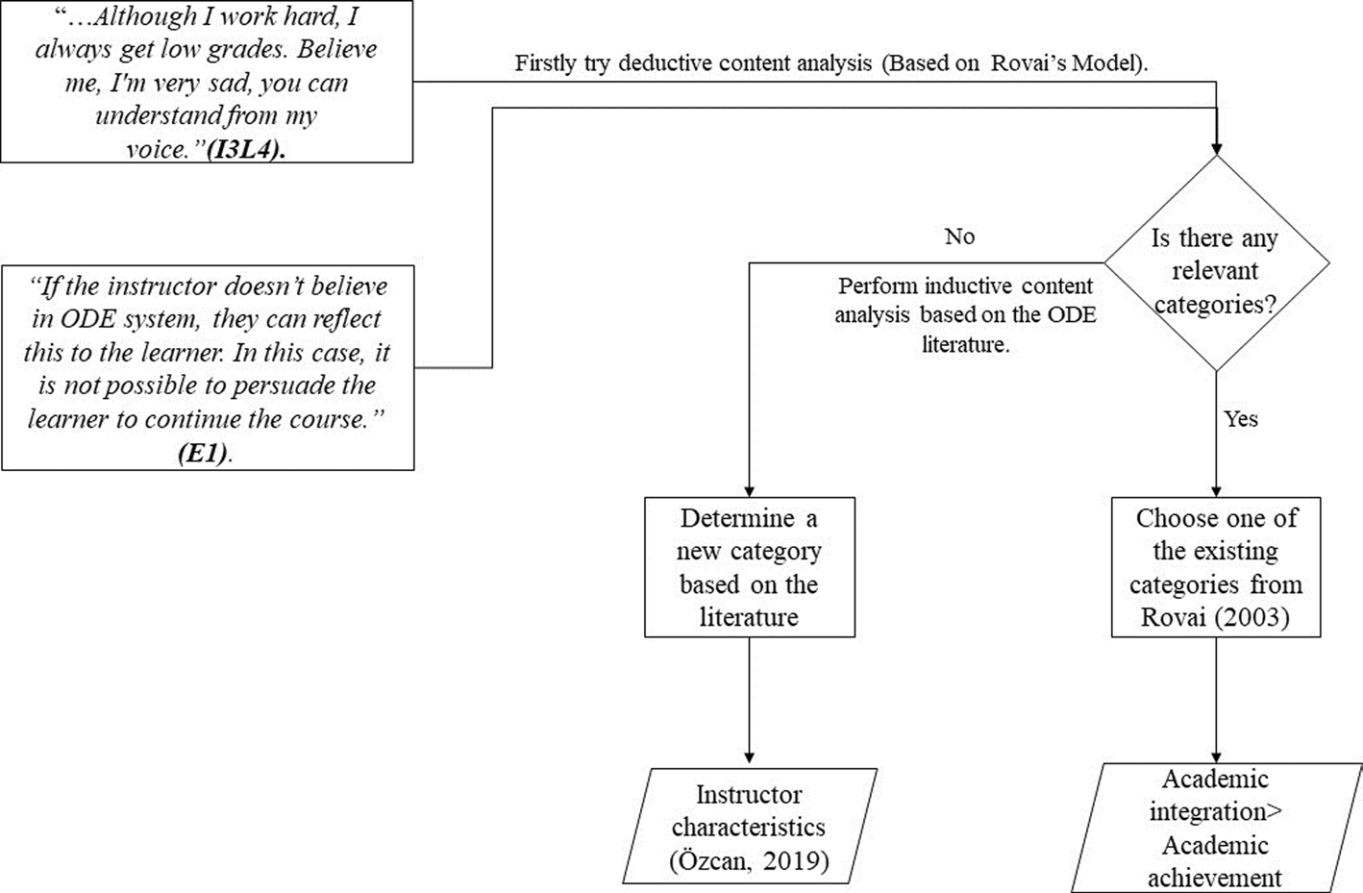
Content analysis process

Content analysis can be done with deductive and inductive methods. Since the structure of the analysis in this study is determined based on the Rovai’s (2003) Composite Persistence Model, it is mostly deductive (Kyngas & Vanhanen, 1999); however, inductive content analysis (Hsieh & Shannon, 2005) was used as some codes, categories, and themes emerged during the analysis of the text. Berg (2001) and Patton (2014) have stated that in cases where deductive data analysis is not sufficient, new codes and categories can be created with inductive data analysis. Hence, content analysis was conducted deductively based on Rovai’s Composite Persistence Model (2003). Accordingly, new codes and categories were added to Rovai’s model with inductive data analysis. Figure 2 illustrates the content analysis steps for a more comprehensive understanding. Newly added items to Rovai’s model are marked with the asterisk (*) sign in Table 1.

**Table 1:** Dropout reasons

Theme	Category	Code frequencies based on participant groups											
		Student		Field Expert		Instructor		Administrator		Support staff		Total	
External reasons	Business life	14^a^	7^b^	5	3	4	3	2	2	1	1	26	16
	Financial reasons	13	10	4	3	3	3	3	3	3	3	26	22
	Family life	9	5	2	1	6	3	1	1	3	2	21	12
	External support/obstruction	9	5	5	3	–	–	1	1	1	1	16	10
	Social life*	5	4	–	–	–	–	–	–	–	–	5	4
	Life crises	4	3	–	–	–	–	–	–	1	1	5	4
	Opportunity to transfer	–		4	3	–	–	1	1	1	1	6	5
	Total	54	15	20	5	15	6	9	3	11	6	109	35
Internal reasons	Academic integration	38	16	9	5	4	3	7	3	7	4	65	31
	Social integration	32	12	19	3	4	2	5	2	7	3	67	22
	Resources*	26	9	3	3	–	–	2	2	2	2	33	33
	Accessibility	24	9	14	4	3	3	5	3	11	7	57	57
	Instructor characteristics*	16	6	34	5	18	6	7	3	9	2	84	22
	Program compatibility	15	6	3	3	–	–	1	1	4	3	23	23
	Utility	11	5	2	1	2	1	3	2	2	2	20	20
	Exams*	8	6	–	–	4	3	2	2	3	1	17	12
	Perceived ease of completion*	6	3	2	1	1	1	6	4	9	3	24	12
	Institutional commitment	5	4	2	1	2	1	1	1	–	–	10	7
	Absenteeism	3	2	–	–	–	–	–	–	–	–	3	2
	Anxiety	3	1	–	–	–	–	–	–	–	–	3	1
	Course availability	2	2	2	2	–	–	–	–	1	1	5	5
	Flexibility*	2	2	5	2	–	–	–	–	–	–	7	4
	Program fit	2	1	–	–	–	–	1	1	1	1	4	3
	Orientation*	2	1	2	2	–	–	–	–	–	–	4	3
	Diploma validity	1	1	–	–	1	1	2	2	1	1	5	5
	Motivation*	1	1	–	–	–	–	–	–	–	–	1	1
	Satisfaction	–	–	6	3	–	–	–	–	–	–	6	3
	Total	198	17	104	5	39	6	42	4	59	8	440	40
Student skills	Digital literacy	5	3	5	3	–	–	2	1	2	2	14	9
	Self-regulation*	3	2	6	4	–	–	2	2	2	2	13	10
	Total	8	5	12	5	–	–	4	2	4	4	28	16
Student characteristics	Personality structure*	7	5	5	3	1	1	3	2	–	–	16	11
	Study habits	4	3	1	1	1	1	1	1	2	2	9	8
	Goal commitment	3	2	2	1	5	2	3	2	5	5	18	12
	Belief/preconception*	3	2	1	1	–	–	2	1	1	1	7	5
	Age	3	2	–	–	1	1	–	–	–	–	4	3
	Self-suitability*	1	1	–	–	–	–	1	1	–	–	2	2
	Academic background	1	1	2	2	3	2	–	–	1	1	7	6
	Un/consciousness*	–	–	2	1	–	–	5	4	4	3	11	8
	Technical equipment facilities*	–	–	–	–	–	–	1	1	–	–	1	1
	Total	22	9	15	5	11	4	16	4	13	6	77	28
General total		281	17	151	5	65	6	71	4	87	8	654	40

*Newly added items to Rovai’s composite persistence model (2003)

^a^The number of times the code was repeated

^b^The number of participants indicated the code

First, interview data are transcribed for analysis. To ensure coder reliability, 7 of the 40 transcripts were analyzed by a different open and distance education expert. As a result, inter-coder agreement (Reliability = consensus/consensus + disagreement) was calculated as 90.29%, which Miles and Huberman (1994) find 80% as a good fit.

### Researchers’ role

The researchers have extensive experience in the field of distance education with their dissertations focused on distance education. One of the researchers was the director of the distance education center of one of the largest universities at the time the study was conducted and she instructs courses on distance education at the undergraduate, graduate and doctoral levels. The other researcher has enrolled in many courses related to distance education at undergraduate, graduate and doctoral levels. Both researchers have academic studies (books, articles, proceedings) on student engagement and interaction in open and distance learning. Researchers have experienced and competent in qualitative studies and data analysis. All this experience and knowledge has positively affected the researchers’ conduct of this study and interpretation of the data.

## Findings

In the study, factors that cause ODE students to drop out are evaluated from the perspective of students, ODE experts, administrators, instructors and support staff. Table 1 shows the reasons for students to drop out from the perspective of the participating groups separately and in a holistic manner.

Table 1 shows that internal reasons, external reasons, student characteristics and student skills are effective in dropping education, respectively. This means that students associate the dropout reasons more with the period after admission. Considering the opinions of other participants and the general opinion, it is seen that the ranking is compatible with the students. However, it is worth emphasizing that the instructors did not mention student skills at all.

It is necessary to interpret Table 1 based on Rovai’s model before interpreting it according to participant views. The categories indicated by the * sign in Table 1 are the categories that were not included in the model and were added as a result of the research. In this study, the places and/or names of some categories were changed. For example, “study habits and goal commitment” are among the internal factors in the Rovai’s model. However, as a result of this study, and after consulting expert opinions, it was decided that it would be more appropriate to include these categories under student characteristics. In addition, the variables considered as “computer literacy and computer-based interaction” in Rovai’s model are combined here as “digital literacy” in accordance with the current literature. The addition of new categories such as “instructor characteristics, resources, exams”, which are not included in Rovai’s model, to the category of internal reasons can be interpreted as changing students’ expectations over the years, depending on the technological opportunities. Another point to be noted is that variables such as “ethnicity, and gender” in Rovai’s model were not observed in the results of this study. The categorical changes in the study did not create a new model; Rovai’s model was used as a sound basis.

### Internal reasons

According to student views, the most influential internal factors are academic integration, social integration, resources, and accessibility. However, field experts highlighted the features of the instructors, social integration (mostly student–instructor interaction) and accessibility. The instructors, like the field experts, mostly emphasized the instructor characteristics, and the support staff focused mostly on accessibility, which is the focus of their tasks.

#### Academic integration

Academic success, being able to follow the courses, the difficulty of the courses and the number of courses enrolled are directly related to academic integration as stated: “…*Although I work hard, I always get low grades. Believe me, I’m very sad, you can understand from my voice.” ****(I3S4)***.

#### Social integration

One of the significant categories for internal reasons is social integration that includes student–student interaction, student–instructor interaction, and presence in the campus environment. The statement *“…It is not easy to adapt and focus in distance education. It is difficult for a person to do something alone. It’s easier when it’s with the community.”* ***(I4S4)*** indicates that the student needs to interact with other students. A student reported his search of a campus environment with the following sentences: *“So I would eat pasta. I would be hungry, there would be no pocket money… I would live the classic life of university students.”****(I4S3)***. The effect of student–instructor interaction can be seen with the following statement: *“We had an instructor who was never concerned with our problems. He did not help us when we called.”****(I4S2)***.

Social integration was also frequently addressed by other participants. “*Of course, it is very important that the instructor is accessible. There is a huge difference between a student who knows that he will receive a response from the instructor when he sends an email and a student who knows that he will not receive a response for days.”***(FE4)**. “*Students usually complain about the instructor not coming to the live sessions on time. Although the instructor comes on time, he does not start the lesson on time. He keeps the students waiting, takes long breaks.”****(I1SS2)***.

#### Resources

Students care about having printed books, and quality and quantity of the resources: *“I must have a book in my hand. I will underline, cross out, and read. They don’t give books anymore.”****(I3S4)***.*“Resources were not sufficient (in number and variety). Since I could not study properly due to lack of resources, I could not pass the lessons.” ****(I4S2)***. However, other participating groups did not focus on resources that students highly care. Instructors and administrators think that course contents and other resources provided by the institution are sufficient. One of the administrators even claimed that the students used the resources as an excuse to leave the institution.

#### Accessibility

Accessibility of the institution is important for students. Students attach great importance to having a close examination center, being able to solve their transactions online, and being able to access educational-technical support whenever necessary. *“One of my reasons for dropping out is that the exams are held in a city far from me…”* ***(I1S4)***. *“One of my reasons for dropping out was that the institution didn’t have the ability to solve transactions online.”* ***(I3S5)***. *“I cannot reach the call center, the music playing while waiting makes me nervous. I say ‘damn it’ and close.” ****(I2S1)***. Field experts and support staff also frequently mentioned accessibility. “*During our busy periods, we cannot respond to the demands of the students. In our call center, there may be periods when the number of working people is not sufficient for the callers.” ****(I2SS2)***.

#### Instructor characteristics

Instructor characteristics are one of the most emphasized features. Students emphasized the digital literacy skills of the instructor: *“One of the most important reasons is that the instructors are technologically insufficient… We say ‘Can you bring the microphone closer?’ and some instructors can't even do that.” ****(I1S4)***. One of the field experts highlighted the instructors’ feature of “believing in ODE” as follows: *“If the instructor doesn’t believe in ODE system, they can reflect this to the student. In this case, it is not possible to persuade the student to continue the course.”* ***(FE1)***. The instructor’s belief in ODE has also been emphasized by the instructors themselves: *“The instructor who does not believe in ODE teaches involuntarily. It would be better if they do not attend that class. Because the students feel it.”* **(I1Ins2)**. While instructors were expected to be aware of the student problems during synchronous lessons, instructors were the group that mentioned the categories specified by the students the least.

#### Program compatibility

Students’ failure to choose the program in line with their interests and their inability to adapt to the program are also among the noticeable factors in dropping out: “*If I had chosen the program I wanted, I would never drop out.” ****(I3S4)***. However, apart from the students, only the support staff mentioned program compatibility. Accordingly, it can be said that the other participating groups cannot empathize with students. The same applies to the institutional commitment. It is quite striking that the administrators have almost never emphasized this issue.

#### Utility and exams

Utility and exams are also important to students; however, other participants are not sufficiently aware of these factors. While students care about the program’s provision of financial returns and job opportunities, only six of the other participants named these factors.

#### Perceived ease of completion

Administrators and support staff stated that students expected to complete their education easily, and they drop out if this expectation was not met. *“Students has a perception that ODE is easier. But once they enter the system, they come across a very reliable assessment and evaluation system.” ****(I3A)***.

### External reasons

The second influential factor in the decision to drop out is the external reasons (see Table 1). Students highlighted business life, financial reasons, family life, and external support/obstruction as external reasons.

#### Business life

The intensity of business life led some students to drop out: *“It was exceedingly difficult to study and work. Also, at that time I was working in shifts. Therefore, I could not attend classes anyway.” ****(I4S1)***. Some students may drop out with the comfort of having a job. This idea was supported by a field expert as follows: *“Graduate students are generally working people. If they can’t find what they’re looking for, they don't much choose to persist.” ****(FE2)***.

#### Financial reasons

Financial reasons are the factor mentioned by the most participants. The high tuition fee and/or the financial situation of the student may lead them to decide to drop out. *“The fee was too high. My registration has already been deleted because I could not pay the fee.” ****(I4S1)***. *“The most prominent reasons for dropout are the economic reasons.” ***(I4SS2)**.

#### Family life

One of the reasons for dropping out is family life. The responsibility of children is an important obstacle for female students: *“Studying with children is difficult. The child’s sleep time, eating time, and so forth can become a problem. You have to stop studying. This is actually a reason for dropping out.” ****(I3S2)***.

#### External support/obstruction

Sometimes, a student’s social environment and family may prevent her/him from continuing the education. The statements of **I3S4** are quite striking: *“Especially my husband said: ‘Leave that school! You don’t understand anyway, you fail the lessons… You can’t succeed. You work, but you can’t do it…’”* However, the participants other than the students did not pay much attention to these factors. It was found that especially the administrators and the instructors were not sufficiently aware of the factors other than the educational life of the students and could not understand the students’ conditions.

### Student characteristics

Students’ characteristics such as personality structure, study habits, goal commitment, and beliefs/preconception about ODE are effective in the drop out decision (see Table 1). Other participants as well as students thought that student characteristics are important, too. Still, when looking at the number of participants and the frequency of the codes, it is seen that the other participants do not have enough empathy with the students. It can be said that the most successful group in this regard is field experts and support staff. According to the students, factors such as personality structure, study habits such as not being able to work with e-resources, not being committed to the goal enough, not believing in ODE are the most important student characteristics that lead them to drop out. Some striking categories were explained under the following headings.

#### Personality structure

***I3S5*** stated that she has dropped-out due to her perfectionism (personality structure): *“I would have completed open education much more easily. I could not finish it because I was trying to get high marks. I was already passing the exams.”*

#### Study habits

The following statement of ***I2S2*** reveals the importance of being able to work with e-resources (study habits): *“The institution does not have any printed books. Working from the e-book is very tiring for my eyes. I wouldn't have chosen this institution if I knew it was like that.”*

#### Prior knowledge

One of the field experts ***(FE5),*** emphasized the importance of prior knowledge as follows: *“A student with insufficient prior knowledge will have difficulty understanding the subject. Maybe this will lead to failure and then to dropping out.”****I4Ins3***, on the other hand, discussed goal commitment as follows: *“They must have clear goals. A person who has no goal will not continue anyway…”.*

#### Un/consciousness

The administrators emphasized the unconsciousness of the students the most: *“The student doesn’t know what a problem is with not being able to watch the recording. For example, the instructor may not have taught the course yet, the student is persistently looking for the recording. But there is no recording. Here, the unconsciousness of the student is the main problem.”****(I1A)***.

### Student skills

Student skills have been the least emphasized theme. Participants believe that students with insufficient digital literacy and self-regulation skills tend to drop out. It is surprising that the instructors never mentioned student skills.

#### Digital literacy

A student expresses her problem with digital literacy as follows*: “I couldn't attend any of the classes. I am very clumsy about technology. I always tried to manage with what friends wrote and sent *via* WhatsApp.”* ***(I1S1)***. A support staff referred to digital literacy as follows: *“Technical inadequacy of students can sometimes cause them to drop out. For instance, we have students who say ‘I do not want to install any application on my phone, I cannot deal with it, and drop out.” ****(I4SS2)***.

#### Self-regulation

***I3S1 ***explained the effect of insufficient self-regulation skills as follows: *“I’m not a much-organized person either. I was like that in face-to-face education anyway… Unfortunately, I always put things off. This is my philosophy of life.” ****I1A*** mentioned the similar issue as follows: *“It seems to students that everything is simple and easy in ODE; but it requires a serious effort to be honest. It requires good planning and good time management.”*

## Discussion

This study revealed that the educational process especially after enrollment, is extremely important in students’ decision to drop out. One of the most significant factors in this process is the qualifications of the instructor. If the instructor believes in ODE, has field knowledge, cares about the courses, and has ODE experience, students will be more likely to persist. Özcan (2019) determined that some instructors teach in ODE although they do not believe it. On the other hand, according to Hunt et al. (2014), there may be many reasons that encourage instructors to teach in ODE, and determining these reasons necessitate for the effectiveness of ODE.

In relation to the qualifications of the instructor, the interaction with the students influences the decision to drop out. Feedback given by the instructor highly affects the students that confirms the research results of Shikulo and Lekhetho (2020). Yuan and Kim (2014) also stated that the lack of student–instructor interaction may cause students to feel isolated and drop out. Although Alberti and Pereira (2018) stated that this interaction was ineffective, the importance of student–instructor interaction was appreciated by many researchers, including Moore (1993) and Garrison (2007).

Students need to interact with other students for academic and/or social purposes. Students’ engagement in peer interaction contributes to learning. Student–student interactions lead to learning from each other as emphasized in various studies (Bawa, 2016; Muljana & Luo, 2019; Stone & O’Shea, 2019).

Not being able to obtain academic contribution is one of the reasons leading to dropout. In addition, students’ incompetence to follow synchronous sessions is one of the obstacles to academic integration. It was also determined by Aydin et al. (2019), Cochran et al. (2014), and Sorensen and Donovan (2017) that academic failure leads to drop out decision.

Students care about the accessibility of the services/institution. The most important factors stated by students—the proximity of the examination center to the student and the online accessibility of the institution (online registration, online tuition payment, etc.) are not among the common factors in the literature. Research (Muljana & Luo, 2019; Netanda et al., 2019) considers accessibility as providing technical support. However, the world has recently experienced the importance of online accessibility of the institution especially in the COVID-19 pandemic.

The problems associated with quality and quantity of resources such as finding the learning material inadequate (Okur et al., 2019) or difficult (Sorensen & Donovan, 2017) may prompt drop out. Choi and Kim (2018) also indicated that insufficient student-content interaction may be a reason for dropping out.

Another factor affecting students’ decision is program compatibility. Students’ assumptions about the effortlessness of ODE (Aydin et al., 2018; Bawa, 2016) might hinder their retention. Some students register without having enough information about the program and then drop out. This finding is consistent with the results of the study conducted by Aydin et al. (2019). Students’ lack of interest in the program may lead to drop outs (Peck et al., 2018).

The students value that the program provides personal and professional development and financial return. The perception of program effectiveness can lead to dropping out. Peck et al. (2018) pointed out that students concentrate on the long term benefit of learning rather than learning itself.

The role of exams, which is one of the factors not included in dropout models, has been revealed in this study. The availability of alternative exams (i.e., make-up exam), reliability, validity, difficulty of exams are imperative to students. Okur et al. (2019) specified that exam conditions are effective in students’ drop out decisions.

In the post-enrollment period, variables other than education may affect dropout behavior. The external reasons determined in this study correspond to the factors in Rovai’s Composite Persistence Model (2003) and previous models. The responsibility of their children, and family life affect mostly the female students. The effects of professional and family life were also discussed by Aydin et al. (2019), Sorensen and Donovan (2017), and Lakhal and Khechine (2021). Besides “external obstacle,” is one of the external reasons that was stated in the studies of Park and Choi (2009), and Hart (2012).

The leading factors determining student persistence seem to be the goal commitment and personality structure. Personality structure is not encountered in dropout models. Goal commitment was discussed in Tinto’s (1975) Student Integration Model. According to Okur et al. (2019), students who do not have an academic or professional career expectation, may drop out easily. Aydin et al. (2019) indicated that the career goals of the student can be determinant in dropout behaviors.

In this study, the importance of students’ “personal characteristics” such as perseverance, patience, and ambitiousness, which are not included in dropout models, have been investigated. Lack of self-confidence, perfectionism, impatience, and lack of determination are found to be possible dropout reasons.

Finally, it has been observed that students’ self-regulation and digital literacy skills may have an impact on their dropout behavior. Self-regulation has similarly been emphasized by Aydin et al. (2019), Bawa (2016), and Stiller and Bachmaier (2017). Furthermore, students’ incompetence to use technology for educational purposes may cause them to drop out (Bawa, 2016).

## Conclusions, limitations, and suggestions

Many studies emphasized that dropout rates in ODE are increasing (Muljana & Luo, 2019; Radovan, 2019; Xavier & Meneses, 2020). Numerous studies attempt to determine the reasons for dropping out and the measures to be taken (Aydin et al., 2019; Peck et al., 2018). Consistent with the literature, the findings of this study shows that internal and external factors lead students to the decision to drop out. Additionally, student characteristics and student skills can affect students’ decision. Stakeholders are found to be not effectively empathizing with students. The stakeholders are expected to analyze their students at the very beginning and throughout the process, understand student characteristics, skills and expectations, and shape the activities accordingly.

The study has many important implications that might be useful to practitioners and all related stakeholders; however, only some of the prominent recommendations are presented here.

Considering the importance of academic success in the retention of students, it should be examined and students in the risk group should be determined. Students highly value the benefit of social interaction. In regard to this, social media groups and, where possible, face-to-face social activities should be organized to ensure and maintain student–student interaction. In addition, the instructor plays a key role for the students. It can be recommended that instructors and administrators should be evaluated and employed according to the predetermined criteria such as ODE knowledge, experience, vision, administrative skills, and leadership. Besides, instructors should be trained about the theoretical background of ODE and student engagement. Measures should be taken to guarantee student-instructor interaction in online or face-to-face meetings. Students’ need of resources and the quality of these resources deserve attention. For this reason, wide range of resources should be provided, and the quality of these resources should be confirmed by a board of experts. The students’ focus on the accessibility of the institutions calls for institutions to reconsider their availability and accessibility (i.e. call centers, online support and examination centers). Finally, it is necessary to obtain in-depth information about student needs during online or face-to-face meetings, and monitor the dropout rates.

While this study approaches the problem from a broad perspective, it has its limitation. In this direction, some suggestions are provided to the researchers. In this study only the assistant administrators could be interviewed since the managers were very busy. Future studies can obtain more comprehensive data by including the administrators. Another suggestion is to conduct mixed studies using scales about the significant themes highlighted in this study. The results of qualitative studies are not generalized. However, the objectivity of the researchers and the reliability of the analysis, and data analysis based on a sound model and literature, similar results could be expected in alike studies. On the other hand, due to the dynamic structure of open and distance education, it is subject to critical changes, especially as a result of COVID-19, diverse results are likely to be achieved. Researchers are recommended to analyze the situation based on their own geographical and cultural context, and profiles of the related regions, institutions.

## Data Availability

Authors may share data and material upon request, provided that ethical principles are respected.
